# Effect of crocin on nitric oxide synthase expression in post-ischemic isolated rat heart

**Published:** 2015

**Authors:** Mahdi Esmaeilizadeh, Mahin Dianat, Mohammad Badavi, Alireza Samarbaf-zadeh, Bahareh Naghizadeh

**Affiliations:** 1 *Physiology Research Center, Faculty of Medicine, Ahvaz Jundishapur University of Medical Sciences, Ahvaz, Iran*; 2 *Physiology Research Center and Department of Physiology, Faculty of Medicine, Ahvaz Jundishapur University of Medical Sciences, Ahvaz, Iran*; 3 *Department of Virology, Faculty of Medicine, Ahvaz Jundishapur University of Medical Sciences, Ahvaz, Iran*; 4 *Department of Pharmacology, Faculty of Medicine, Ahvaz Jundishapur University of Medical Sciences, Ahvaz, Iran*

**Keywords:** *Ischemia-reperfusion injury*, *Crocin*, *Vitamin E*, *iNOS protein*, *eNOS protein*, *Rat*

## Abstract

**Objective:**

Oxidative stress damages cells and brings about the pathogenesis of ischemia/reperfusion injury. This study was carried out to investigate the preconditioning and cardio protective potential effects of crocin and vitamin E by the eNOS and iNOS express gene in ischemia/reperfusion in rats.

**Material & Methods:**

Male rats were divided into seven groups, namely: sham, control group and experimental groups treated with crocin(10, 20 and 40 mg/kg), vitamin E (100 mg/kg) and combination of crocin (40 mg/kg) with vitamin E (100 mg/kg) that were gavaged The heart was removed and relocated to a Langendorff apparatus and subjected to global ischemia and then the left ventricular end diastolic pressure (LVEDP) were measured as a hemodynamic parameter. Total RNA was extracted from heart frozen tissues. RT-PCR technique was performed by specific primers designed for nitric oxide gene and the results were assessed by agarose gel electrophoresis.

**Results:**

Results after ischemia and reperfusion showed that crocin 40 mg/kg produced a significant improvement of LVEDP as a mechanical function (p<0.05), associated with a reduction of iNOS release (p<0.05). The eNOS mRNA levels were significantly higher in crocin-treated 40 mg/kg compared to controls treated by RT-PCR technique. The combination of crocin and vitamin E have shown more effective on the reduction of iNOS release (p<0.01).

**Conclusion:**

In the isolated rat heart, protective effect of crocin, may possibly be explained by regulating eNOS and iNOS expressions. The Results resultsconfirmed the hypothesis that cardioprotective effect of crocin is partly mediated by nitric oxide. This could explain the cardioprotective action of crocin following ischemia and reperfusion.

## Introduction

It has been shown that early reperfusion is effective in preventing cell death after coronary artery ischemia. However, the blood flow restoration leads to harmful complications and cause damage that has been termed reperfusion injury. This may affect various aspects of myocardial and endothelial function, with complex pathophysiological consequences (Lopaschuk et al., 2003 ▶). Myocardial injury after ischemia reperfusion (I/R) require invasive interventions such as coronary bypass surgery and angioplasty to reestablish coronary blood flow and reduce cardiac damage due to severe myocardial ischemia (Jahanbakhshet al., 2012 ▶). It is now recognized that reentryof oxygenated blood into ischemic myocardium can initiate a cascade of events that will paradoxically produce additional myocardial cell dysfunction (Boyleand Weisman, 1993 ▶) and this is associated with changes in hemodynamic, electrophysiology and antioxidant capacity (Osada et al., 1998 ▶).

*Crocus sativus L*., traditionally known as saffron, is used for several purposes in folk medicine and contains carotenoid pigments, bitter glycoside and an aromatic substance. Crocin, one of the active components of saffron, has the carotenoid pigments ester structure (Hosseinzadeh and Ziaei, 2006a ▶). Pharmacological and clinical studies have suggested that crocin can be used as a novel therapeutic factor such as antioxidant (Dianat et al., 2014a ▶), antitumor (Fernandez, 2006 ▶) and radical scavenging (Assimopoulou et al., 2005 ▶). The cardio protective effects of saffron and crocin have been reported in some studies in relation to action of modulating of endogenous antioxidant enzymatic activities (Hosseinzadeh, et al., 2009b ▶; Jahanbakhshet al., 2004 ▶). In addition, crocin has been shown to have antioxidant effects in ischemia-reperfusion models of stroke in rat brains (Ochiai et al., 2007 ▶). It has been reported that isolated rat hearts subjected to Global I/R experience some changes in hemodynamic factors such as reduce left ventricular systolic pressure (LVSP), heart contractility (±dP/dt), and increased left ventricular end diastolic pressure (LVEDP) (Jahanbakhshet al., 2012 ▶). On the other hand, vitamin E is an exogenous radical scavenger. It has been stated that vitamin E prevents the suppression of ventricular function in the rat (Dhalla et al., 2000 ▶). The nitric oxide regulates several important aspects of vascular responses associated with the lack in the amount of bio available vascular NO, resulting in endothelial dysfunction (Atochinand Huang, 2010 ▶). Regulation of NO production requires both bases and motivated production of NO by eNOS enzyme utilizes L-arginine as substrate. NO is manufactured in the heart by eNOS present in the cardiac myocytes and endothelium. Endothelial production of NO is critical to the regulation of vascular responses, including regional blood flow, vascular tone, and platelet aggregation (Shaul, 2002 ▶). Due to deficiency in the amount of NO which results in endothelial dysfunction, this study focuses on rat model of ischemia reperfusion caused by direct genetic modification of the endothelial nitric oxide synthase (eNOS) and inducible nitric oxide synthase (iNOS) genes. Some studies have shown that combination of vitamins with other antioxidants produce synergistic effects. Evidence indicates that crocin has a synergic effectwith α-tocopherol on low density lipoproteins in human (Zhou et al., 2005 ▶). Therefore, the current study assessed the effect of the combination of crocin and vitamin E on nitric oxide synthase expression in post-ischemic cardio protection. 

## Material and Methods

**Chemicals**

Vitamin E (Purity: 98%) from Sigma–Aldrich Co. (St. Louis, MO, USA) andcrocin(Purity: 98%) wereobtained from Fluka (Japan), and Krebs salts from Merck Co. (Germany). 

**Animals**

 Male Sprague-Dawley rats (200-240 g) were usedand kept under controlled conditions of temperature at 25±2 ^◦^C, relative humidity of 60±5% and light: dark cycle of 12h. The animals were fed chow pellets and allowed water ad libitum. The examination was approved by the Animal Ethics Committee of Ahvaz Jundishapur University of Medical Sciences (No. ajums.REC.1392.90, Date: 4.05.2013), Ahvaz, Iran.

**Experimental groups**

Seventy malerats were randomly divided into seven groups, including: sham, control and experimental groups treated with concentrations of crocin(10, 20 and 40 mg/kg), vitamin E (100 mg/kg) and a combination of crocin (40 mg/kg) with vitamin E (100 mg/kg). In treatment groups, the rats were orally administered crocin once a day for 3 weeks (Upaganlawar and Balaraman, 2010 ▶) and vitamin E for 30 days (Srivastava et al., 2012 ▶). In treatment groups, crocin and vitamin E were gavaged.

**Experimental protocol**

**Figure1:**
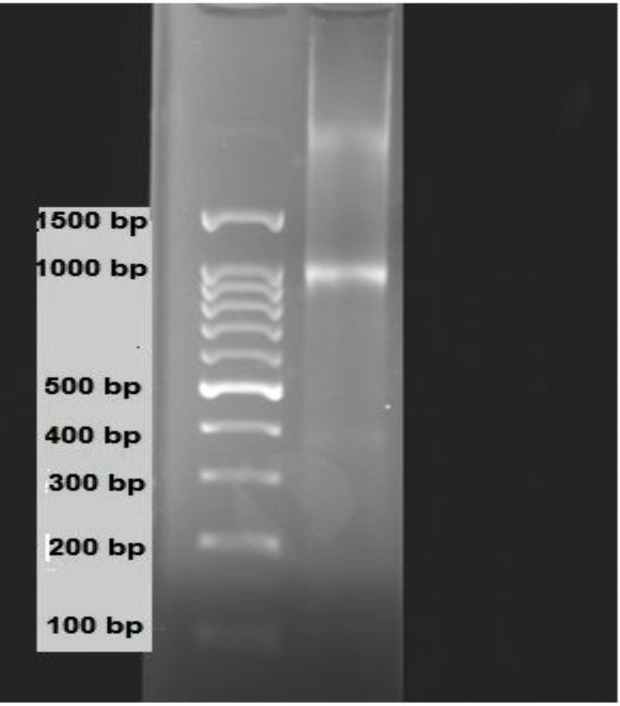
Spectrophotometerimage of total RNA, extracted from frozen hearts was run in 2% agarose gel. 1 ml Trizol + 100 mg tissue. OD: 260/280, C: 1.8-1.9

The rats were anesthetized by Ketamine HCL (50 mg/kg), xylazine (5 mg/kg). The trachea was cannulated and the animals were ventilated using a rodent ventilator. After opening the chest; a steel cannula was placed into the aorta and the heart was quickly removed and transferred to a Langendorff apparatus while perfuse with Krebs Henseleit buffer (NaCl115 mM, KCl4.6 mM, KH2PO4 1.2 mM, MgSO4 1.2 mM, CaCl2 2.5 mM, NaHCO3 25 mM, glucose 11 mM) continuously and equilibrated with 95% O2+5% CO2, PH of 7.4 at constant pressure of 70 mm Hg (Dianat et al., 2014b ▶). Left ventricular pressure was measured with a latex balloon inflated to a diastolic pressure of 5–10 mmHg. The cardiac parameter, left ventricular end diastolic pressure (LVEDP), was monitored by a PowerLab method (ADInstruments, Castle Hill, Australia) (Garcıa-Villalon et al., 2009 ▶). The heart were perfused for 25-30 min before the induction of ischemia to allow stabilization, and then subjected to 30 min of no flow ischemia followed by 60 min of reperfusion. After reperfusion, the hearts quickly excised, fixed in liquid nitrogen, stored at 80 °C and then homogenized for analyses. Quantitative reverse-transcribed polymerase chain reaction (RT-PCR) was used to determine levels of the nitric oxide synthase isoform (iNOS, eNOS) mRNA in rat heart tissues (Chun et al., 2003 ▶; Di Napoli et al., 2007 ▶). Total RNA was isolated as recommended by the manufactures (Figure 1). 

The RNA was dissolved in diethylpyro carbonate (DEPC) water solution and expressed the quantity spectrophotometrical at 260-280 nm. The first strand of cDNA was generated by adding RNA. Glyceraldehyde-3-phosphate dehydrogenase (GAPDH) was used to correct for variation of different samples (Di Napoli et al., 2007 ▶). The PCR solution contained 10 µl of the first strand cDNA, 4 µl 10% PCR buffer, 2mM MgCl2, 0.15mM of both sense (5-ACC ACA GTC CAT GCC ATC AC-3) and antisense (5-TCC ACC ACC CTG TTG CTG TA-3) GAPDH primers, 0.15mM of both sense (5_CGA GAT ATC TTC AGT CCC AAG C-3) and antisense (5-GTG GAT TTG CTG CTC TCT AGG-3) eNOS primer, (Taq) DNA polymerase and water to a final volume of 50 µl. These samples were subjected to 35 cycles at 95 °C for 60 s, 60 °C for 60 s, and to one cycle at 72 °C for 7min (Chun et al., 2003 ▶; Di Napoli et al., 2007 ▶). PCR products were run on 2% agarose gel electrophoresis and photographed under UV light. Bands on the gel were scanned and quantified using a computerized densitometry system (Bio-Rad Gel Doc 1000, Milan, I).

**Statistical analysis**

The results were analyzed using SPSS and expressed as mean ± SEM. Comparisons betweengroups were performed using one way ANOVA for multiple-group comparisons gene expression and repeated measurement ANOVA For hemodynamic analysis, post hoc LSD was used. The (P<0.05) was considered statistically significant. 

## Results

To analyze whether crocin could protect cardiac performance, hemodynamic parameter left ventricular end diastolic pressure (LVEDP) was measured. The marker of preload (LVEDP) elevated during reperfusion, but it decreased by administration of crocin in dose (20 and 40 mg/kg) (P<0.05). The combination of crocin (40 mg/kg) and vitamin E (100 mg/kg) yielded a significant effect (p<0.01).

**Effects on iNOS mRNA expression levels**

**Figure 2:**
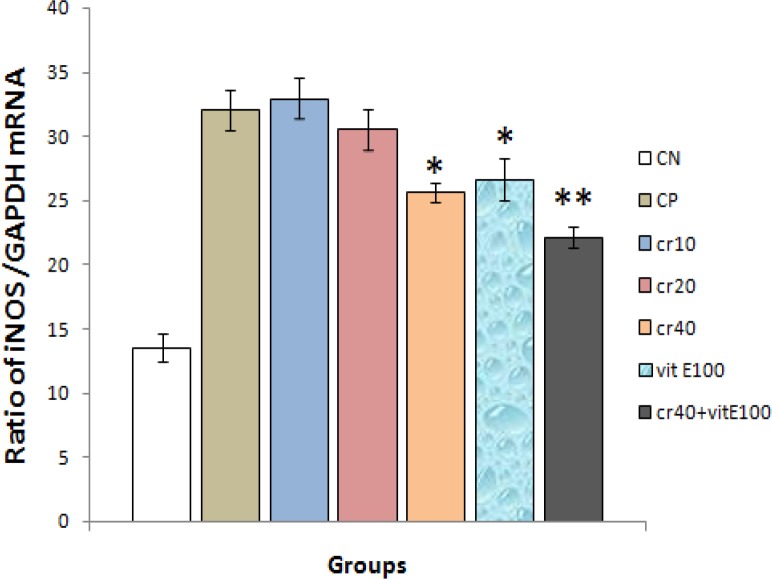
The iNOS amplifications of mRNA ratio GAPDH in different experimental treatment groups by RT-PCR technique. Results are expressed as mean ± SEM of 10 hearts per group, one way ANOVA followed by LSD test was used. *p<0.05, **p<0.01 were compared with control positive group. CN: control negative, CP: control positive, cr: crocin, vit: vitamin

Ischemia–reperfusion induced showed a significant reduction in iNOS mRNA levels. Crocin in doses of 10 and 20 mg/kg did not show a significant increase of iNOS mRNA levels compared with the control groups (Figure 2).

Crocin in dose of 40 mg/kg and vit E 100 mg/kg produced a significant decrease in iNOS mRNA levels compared with the control group (p<0.05). Results of the group of crocin plus vitamin E did significantly affect the iNOS mRNA levels (p<0.01). GAPDH mRNA levels are used for densitometry formalization of iNOS mRNA levels. 

**Effects on eNOS mRNA expression levels**

**Figure3:**
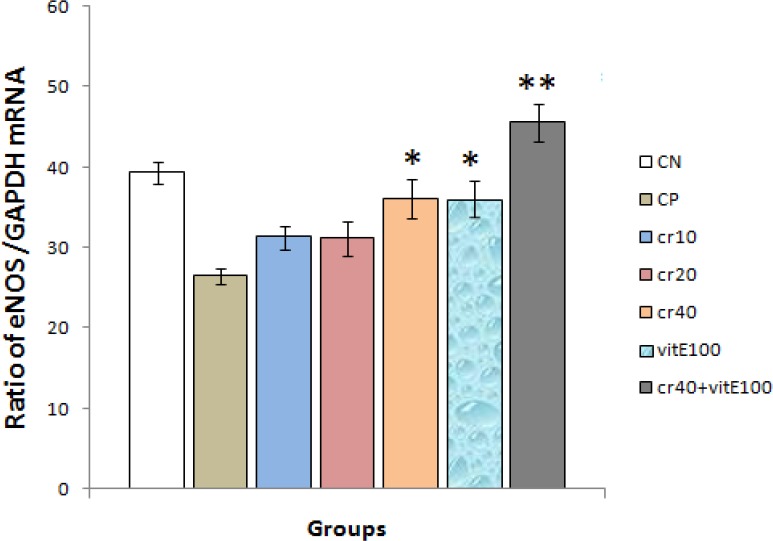
The eNOS amplifications of mRNA ratio GAPDH in different experimental treatment groups by RT-PCR technique. Results are expressed as mean ± SEM of 10 hearts per group, one way ANOVA followed by LSD test was used. *p<0.05, **p<0.01 were compared with control positive group. CN: control negative, CP: control positive, cr: crocin, vit: vitamin

Ischemia–reperfusion induced showed significant augment in eNOS mRNA levels. Crocin in dose of 40 mg/kg produced a significant increase of eNOS mRNA levels compared with control hearts (p<0.05). Results of the group of crocin puls vitamin E did significantly affect the eNOS mRNA levels (p<0.01). Crocin in doses of 10 and 20 mg/kg did not show a significant increase of eNOS mRNA levels compared with control group (Figure 3). The electrophoresis pictures of control and treatment groups shown in (Figure 4A, B).

**Figure4A:**
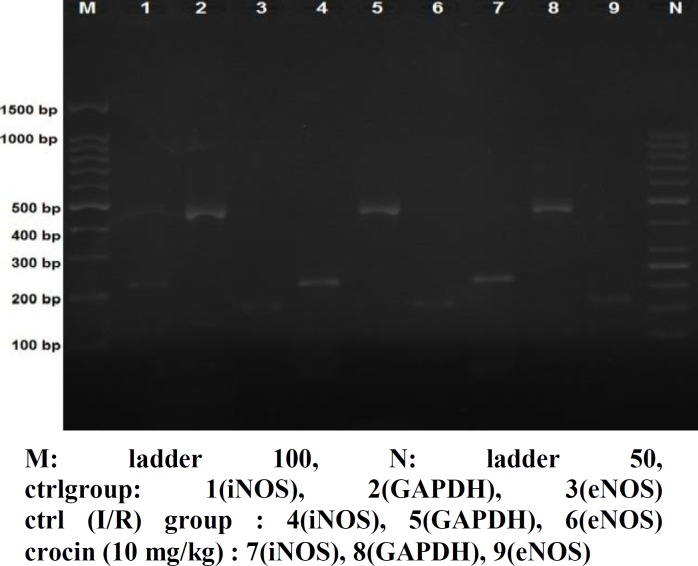
Comparison of eNOS, iNOS and GAPDH, electrophoresis in CP, NP and crocin (10 mg/kg) groups. CN: control negative, CP: control positive, cr: crocin, vit: vitamin

**Figure4B:**
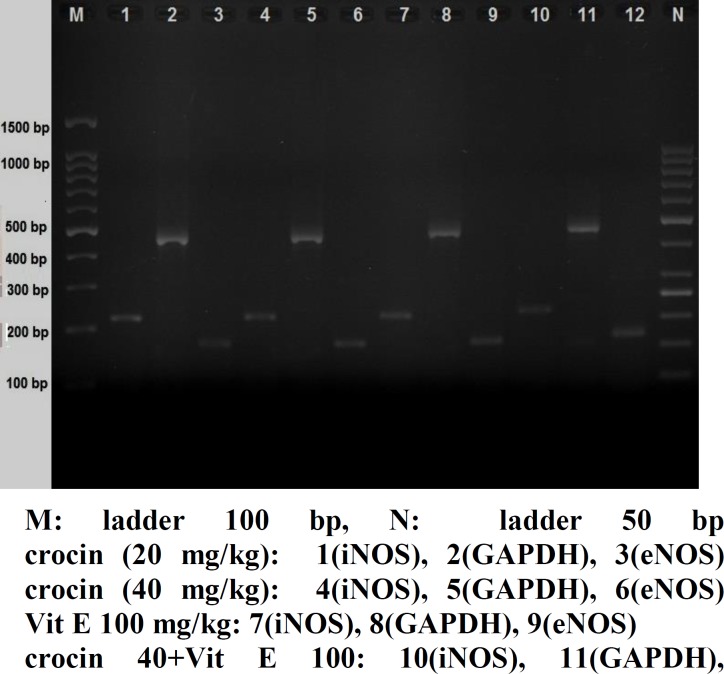
Comparisone of eNOS, iNOS and GAPDH, electrophoresis in crocin (20, 40 mg/kg), vit E (100 mg/kg) and combination of crocin (40 mg/kg) with vit E (100 mg/kg) groups. CN: control negative, CP: control positive, cr: crocin, vit: vitamin

## Discussion

The aims of this study were to determine the protective effects of crocin and vitamin E on myocardial function and NOS expression during global ischemia and reperfusion. The results showed that in isolated rat heart model on I/R administration of crocin reduces myocardial dysfunction and prevents myocardial damage. The global ischemia subsequent to reperfusion in isolated hearts was associated with disturbance of contractile function as pointed out by decreased left ventricular systolic pressure (LVSP), impairment of myocardial contraction force (±dP/dt), and raised left ventricular end-diastolic pressure (LVEDP). 

The elevated LVEDP resulted in sub endocardial low perfusion that makes ischemic injury worse (Goldspink et al., 2004 ▶). Crocin significantly preserved hemodynamic and maintained ventricular functions.The LVEDP decreases due to improved contractility of the heart and resulted in an increase in the subendocardial blood flow (Abdullaev FI and Espinosa-Aguirre JJ, 2004 ▶) and this is possibly related toits antioxidant properties or its effects on NOS synthase. In similar experimental conditions, the beneficial effects of crocin on cardiac hemodynamic were previously reported (Dianat et al., 2014b ▶); however, along with these beneficial effects, we demonstrated that crocin also determines a significant myocardial integrity and function by the simultaneous treatment with vitamin E by affecting NO levels. The anti-ischemic effect of crocin, which can be observed in the absence of any hemodynamic action before ischemia, improves post-ischemic recovery during reperfusion. The beneficial effects of crocin on the I/R mediating free radicals and inflammatory reactions are proved (Dianat et al., 2014a ▶). The effect of the ischemic cells on metabolism are associated with conservation of eNOS and decreased post ischemic hyper permeability, and supply release or increased bioavailability of nitric oxide (NO) appears to be principal. Actually, besides its known vasodilator effects, NO, reduces post-ischemic hyper permeability, decreases platelet adhesion and aggregation, and reduces leukocyte adherence and migration. The eNOS existing in the cardiac myocytes producing NO is produced in the heart (Barouch et al., 2002 ▶). It has been reported that eNOS double knockout mice show evidence of ischemic heart disease and left ventricular dysfunction, making them a practical model in human cardiovascular disease (Kuhlencordt et al., 2001 ▶). on the other hand eNOS impressive mice show increased inotropic and lusitropic responses under basic conditions (Barouch et al., 2002 ▶) and also reported in response to isoproterenol (Gyurko et al., 2002 ▶). Moreover, eNOS deficient mice demonstrate enhanced left ventricular dysfunction and post-myocardial infarction remodeling by diminished hypertrophy (Scherrer-Crosbie et al., 2001 ▶). Previous results indicate that the beneficial effects of vitamin E in acute ischemic syndromes occur through the reduction of oxidative stress (Canbaz et al., 2003 ▶). According to our results, if the reduction damage is due to the effect of NO on myocardial efficiency in ischemic conditions on a theoretical field, it is likely that the modulation of endothelial function by crocin (increase of eNOS expression and decrease of iNOS expression) has beneficial effects on ischemia/reperfusion injury because of the hemodynamic protective effects of NO.

In summary, our results have shown that in the isolated rat heart, crocin exerts a NO-dependent cardio protection against ischemia/reperfusion injury and preserves the heart function. 
